# Risk of breast cancer in relation to dietary intake of selenium and serum selenium as a marker of dietary intake: a prospective cohort study within The Malmö Diet and Cancer Study

**DOI:** 10.1007/s10552-021-01433-1

**Published:** 2021-04-29

**Authors:** Ylva Bengtsson, Malte Sandsveden, Jonas Manjer

**Affiliations:** Department of Surgery, Skåne University Hospital Malmö, Lund University, 20501 Malmö, Sweden

**Keywords:** Breast cancer, Selenium, Risk, Smoking, BMI

## Abstract

**Purpose:**

Selenium has been suggested to be protective against breast cancer, but the evidence remains inconclusive. Hence, it is important to further examine the potential protective effect. This prospective cohort study investigates pre-diagnostic selenium intake in relation to breast cancer risk. In addition, we analyze serum selenium as a marker of dietary intake.

**Methods:**

This study includes 17,035 women in the Malmö Diet and Cancer cohort. Dietary assessment and serum samples were collected at baseline (1991–1996). During 344,584 person-years of follow-up, 1,427 incident cases were retrieved. Cox regression analysis examined breast cancer risks adjusted for potential confounding factors. In addition, odds ratios (ORs) were estimated for 1186 cases and an equal number of controls in relation to quartiles (Q) of selenium intake and groups consisting of a combination of intake and serum selenium levels.

**Results:**

No overall association between selenium intake, or a combination of intake and serum levels, and breast cancer risk was found. The adjusted relative risk for breast cancer in selenium intake Q4 versus Q1 was 0.96 (0.83–1.12) (*P*_trend_ = 0.65). Similarly, adjusted the OR for breast cancer in selenium intake for Q4 versus Q1 was 0.97 (0.76–1.23). The kappa value, 0.096 (*p* = 0.001), showed poor agreement between serum selenium and selenium intake.

**Conclusion:**

Our findings suggest that there is no overall association between selenium intake, or a combination of intake and serum levels, and breast cancer risk. Finally, our results showed a poor correlation between estimated selenium intake and serum selenium.

**Supplementary Information:**

The online version contains supplementary material available at 10.1007/s10552-021-01433-1.

## Introduction

The relation between selenium and breast cancer has been intensely studied, but the results are not consistent [1–9]. Selenium is an essential trace element in the human body and is found in the diet [10]. It exerts its functions as the amino acid selenocysteine incorporated into selenoproteins [11]. Many of these selenoproteins are essential enzymes and mediate their actions through various mechanisms ranging from antioxidant activities to anti-inflammatory effects [12]. The potential cancer-preventive effects of selenium have been suggested to be mediated through the antioxidant activity of several selenoproteins, e.g. glutathione peroxidases, selenoprotein p and thioredoxin reductase [13]. In addition, it has been implied that selenoproteins prevent oncogenic activity by preferentially triggering apoptosis in cells with the highest DNA damage [14].

Regarding selenium status and breast cancer risk, several studies conducted in mice and rats have implied that selenium has a role specifically in breast cancer through a chemo preventive effect [15, 16], but the evidence of an association between selenium and breast cancer in humans is inconclusive. While one recent meta-analysis including 8,132 breast cancer cases by Cai et al. (2016) found that high selenium intake, serum selenium, breast tissue and toenail selenium were inversely associated with breast cancer risk [5]. Another Cochrane review (2018) with 1,393 cases showed no relationship between selenium intake, supplemental selenium, plasma/serum and toenail selenium and breast cancer risk [3]. A third meta-analysis by Kuria et al. (2018) found an inverse association between selenium intake, supplemental selenium and overall cancer risk, but had low statistical power regarding breast cancer-specific risk due to the limited number (216) of breast cancer cases [6]. The largest prospective cohort study on selenium intake and breast cancer risk to date, involving 9,487 breast cancer cases, showed no association between selenium intake and breast cancer incidence in postmenopausal women in the USA [9]. Taking into account all the conflicting results from other studies, additional large prospective population-based studies are warranted.

Previous studies on selenium status and breast cancer risk have mostly been made in countries with a relatively high selenium intake [3, 5, 6]. Since the activity of some selenoenzymes tends to plateau at higher serum selenium concentrations [17], it has been suggested that a potential inverse association with breast cancer risk would be seen in populations with low or relatively low selenium status [11, 18]. Selenium in cereals, vegetables and livestock is related to the amount in the soil [10], and it is known that the soil in Sweden and other parts of Europe has low amounts of selenium [19]. It would thus be beneficial to conduct a study in a selenium-low area such as Sweden.

Selenium levels and selenium function may be affected by several factors [20]. High Body Mass Index (BMI) appears to be correlated with lower selenium levels in postmenopausal women [21] and obesity is also a well-known risk factor for breast cancer [22]. Furthermore, smoking has been shown to lower selenium levels due to increasing the inflammatory response and reducing bioavailability [23, 24], making it important to take these two factors into consideration when analyzing selenium in relation to breast cancer.

The Malmö Diet and Cancer Study (MDCS) is a population-based prospective cohort study which includes 17,035 women living in a selenium-low area [25]. A dietary assessment was conducted using an interview-based diet history method, and serum selenium levels were analyzed from samples collected at baseline, and information was available on lifestyle factors such as BMI and smoking [26].

We have previously reported on the association between pre-diagnostic serum selenium and breast cancer risk [2]. The aim of the current study was to examine breast cancer risk related to pre-diagnostic levels of dietary intake of selenium within MDCS. An additional aim was to study dietary intake of selenium in relation to serum selenium levels.

## Material and methods

### The Malmö Diet and Cancer Study (MDCS)

The study is based on women from MDCS, which is a prospective population-based cohort study in Malmö, Sweden, a city with approximately 250,000 inhabitants at the time of inclusion. The main aim of the study was to estimate the potential association between dietary factors and cancer [27].

The baseline examination took place between 1991 and 1996, and every resident of Malmö born between 1923 and 1950 was invited to participate. Subjects were recruited using a personal letter of invitation (active recruitment), and community-directed invitation (passive recruitment). Finally, a total of 28,098 individuals including 17,035 women participated, which correlates to a participation rate of 43% for women [25].

### Baseline examination

The data retrieved from MDCS consists of a baseline examination including a self-administered questionnaire, a dietary assessment, anthropometric variables (height and weight, blood pressure and body fat mass) and blood samples. The latter were drawn from non-fasting subjects and immediately frozen and stored in a biobank at − 80 °C. Blood sampling was collected at baseline while dietary data were collected approximately two weeks later due to the nature of the dietary data collection. The season was decided from the date of dietary data collection.

The questionnaire was self-administered and included questions regarding lifestyle factors, socioeconomic status, medical history, and for women also reproductive history and menopausal status.

As previously described [28], a woman was considered peri-/postmenopausal if she affirmed in the questionnaire that her menstruation had stopped prior to the baseline examination or if she had undergone bilateral oophorectomy. The previous information was not available for around 2% of the women and, in that case, the woman was classified as peri-/postmenopausal if she was ≥ 42 years old at baseline. Women who were not peri-/postmenopausal according to the aforementioned classification were categorized as premenopausal.

Anthropometric measurements were assessed by physical examination [26], and BMI was calculated as kg/m^2^ and classified as “normal” (BMI < 25), “overweight” (25–30) and “obese” (≥ 30).

Education was categorized as “O-level college” (≤ 10 years in school), “A-level college” (≥ 12 years in school), and “university” (at least one year following A-level college) [25].

Alcohol consumption was defined both by the questionnaire as “nothing last year”, “something last year” or “something last month”, and by grouping subjects into tertiles of average daily intake, expressed in grams/day, according to the seven-day menu book. Alcohol consumption within the past year or month but not the past week was categorized as “infrequent alcohol consumption” [29].

### Dietary data

Dietary assessment was performed in line with a modified diet history method, and showed good ranking compared to a reference method consisting of 18 days of weighed food records [30]. In absolute values, the mean intake of selenium for women was 29 ± 9 µg and 28 ± 10 µg, compared to 28 ± 9 µg and 30 ± 12 µg in the reference group. Furthermore, the mean for energy-adjusted correlation between selenium intake measured by the dietary assessment method used in the MDCS and reference was 0.46 at the end of the study period [30].

The method consists of (1) a seven-day menu book for listing cooked lunch and dinner meals, beverages including alcohol, natural remedies and nutrient supplements, (2) a 168-item dietary questionnaire for valuation of meal patterns, portion sizes of regularly eaten food and consumption frequencies, and (3) a one-hour interview containing questions concerning diet history, were data on, e.g. portion sizes and cooking practices of the foods from the food diary where specified in more detail. The reference period of the dietary questionnaire was the preceding year. The interviewer carefully checked that the answers in the seven-day menu book and the questionnaire did not overlap. Based on the portion size estimates and the frequency from the questionnaire and menu book, the mean daily dietary intake was calculated. The food intake was converted into energy and nutrient intakes using PCKost2-93 from the National Food Administration in Uppsala, Sweden, as described previously in more detail [30]. The dietary intake of selenium is expressed as the sum of food intake of selenium and supplemental intake of selenium.

In September 1994, an alteration in the coding procedures of the dietary assessment was implemented with the aim of reducing the interview time. However, the impact of the alterations was shown to be small [31].

### Matching and study population

The end of follow-up was 31st December 2013. The Swedish personal identity number was used to link MDCS data with the Swedish Cancer Registry and identify breast cancer cases. There were 1,186 incident cases diagnosed during follow-up. Prevalent cancer cases at baseline (*n* = 576) were excluded.

The controls were selected based on a preceding nested case–control study from the MDCS on breast cancer and vitamin D, parathyroid hormone and calcium [32]. That study included breast cancer cases diagnosed up to 31st December 2006. There were 764 incident cases, and an equal number of controls were chosen and matched by incidence density matching, using age as the underlying time scale, matching on age at inclusion (± 2 years), menopausal status (pre‐ vs. peri‐/post) and calendar time at inclusion (± 15 days). This resulted in 704 unique controls, and among them, 694 were free of breast cancer up until 31st December 2013. To get an equal number of controls as the cases in our current study (*n* = 1,186), additional controls were necessary. The remainder of the needed controls were randomly selected from a sub cohort in the MDCS, the cardiovascular cohort. The motive for choosing this sub cohort was that the subjects were planned for genotyping in a parallel study. In the cardiovascular cohort, 3,531 randomly selected women passed the baseline examination. Of these, 2,615 remained free from breast cancer up to 31st December 2013. As needed, 492 of them were randomly selected, making a total of 1,186 controls.

### Laboratory methods

As previously described by Sandsveden et al. [2], the saved serum was analyzed by ALS Scandinavia AB, Luleå, Sweden. A total of 262 women lacked the necessary amount of stored serum and therefore could not be included in the analyses. Using NIST traceable single element standard, samples were analyzed on ICP-SFMS (Thermo Element 2). A reference material, Seronorm, from Sero AS, Norway (Lot 0,608,414), was used and analyzed along with the samples. Analyses were conducted with a quantity of 0.15 ml serum that was diluted to 10 ml with an alkaline liquid containing 0.1% NH_3_ and 0.005% EDTA/Triton-X. The detection level was 0.4 ng/ml, and the interbatch coefficient of variation was 3.4%.

### Statistical method

A high absolute energy intake tends to result in a high intake of micronutrients such as selenium [33]. To control for this, the residual method was used.

The study population subjects were ranked into quartiles (Q1, Q2, Q3 and Q4) based on the residual value of their dietary intake of selenium, meaning the residual from a regression model in which the absolute selenium intake is the dependent variable and the total energy intake is the independent variable. Residuals in our study are presented as the median of total dietary intake of selenium.

The quartiles were compared regarding factors potentially influencing breast cancer risk or/and levels of dietary intake of selenium such as age at baseline, sociodemographic factors, socioeconomic factors, lifestyle factors, menopausal status and BMI. The same comparison was made for quartiles in the full cohort and between cases and controls.

The correlation between serum selenium and dietary intake of selenium was analyzed in a cross table, and value of agreement was calculated and presented as a kappa value with a *p*-value. A *p*-value < 0.05 was considered as statistically significant. Likewise, as a sensitivity analysis, a cross table was used to analyze serum selenium and non-adjusted values of dietary intake of selenium.

All women were followed until the scheduled end of follow-up, 31st December 2018, or until they got breast cancer or died. The incidence of breast cancer per 100,000 person-years was analyzed in different quartiles of selenium intake. The corresponding relative risks (RR), with 95% confidence intervals (CI), were estimated using a Cox’s proportional hazard model. In a subsequent analysis, the RRs were adjusted for age, socioeconomic index, education, marriage, number of children, age at first childbirth, age at menarche, use of oral contraceptives, hormone replacement therapy, menopausal status, oophorectomy, smoking, BMI, alcohol consumption, year of inclusion and season of collection of dietary data (as the selenium content in food may vary by season [10]). Moreover, trends over quartiles of selenium were calculated by introducing the quartile number as a continuous variable in the Cox’s proportional hazard analysis.

The Recommended Dietary Intake (RDI) of selenium is 55 ug/day for adults according to the US institute of Medicine [34]. To investigate the association between the RDI of selenium and breast cancer risk in Sweden, a low selenium country, the same analyses were carried out for dichotomized groups defined as a low-level group (absolute selenium intake ≤ 55 ug/day) and a recommended group (absolute selenium intake > 55 ug/day).

Moreover, a case–control model was used for some of the analyses. Using a logistic regression analysis, odds ratios (OR), with 95% CI, were estimated for cases and controls in relation to the quartile’s selenium intake as compared to the first quartile. Furthermore, as a way of strengthening the exposure variable, serum selenium and dietary intake of selenium were combined to identify groups more likely to have a high or low selenium exposure. Serum selenium and selenium intake were dichotomized into low (a merge of Q1 and Q2) and high (a merge of Q3 and Q4). ORs were calculated for groups combining low and high serum selenium and dietary intake of selenium: low serum + low intake, high serum + low intake, low serum + high intake and high serum + high intake. In a second model, the abovementioned possible confounding factors were introduced as covariates.

High BMI appears to be correlated with lower selenium levels [21]. In addition, BMI has been positively associated with postmenopausal breast cancer and, in many studies, inversely associated with premenopausal breast cancer [35, 36]. Furthermore, smoking has been shown to lower selenium levels [23, 24]. Following this, all analyses were stratified for BMI (≤ 25 kg/m^2^ and > 25 kg/m^2^) and for smoking (ex, current and never). Additionally, following the analyses and discovering the pattern of a potential association, all analyses were also done as compared to a merge of Q2 and Q3.

Several methods are available for adjusting energy intake, and a sensitivity analysis with another statistical approach was performed. This approach involved including total energy intake in the multivariate logistic regression model along with total intake of selenium and the other abovementioned factors. We also performed sensitivity analyses, adjusting for the interviewer who conducted the diet history interview and dietary method before and after September 1^st^, 1994, but not adjusting for baseline year. An additional sensitivity analysis excluded all cases diagnosed within two years following baseline.

The statistical analyses were performed using SPSS version 25.

## Results

### Baseline characteristics

Compared to the controls, the cases were younger at the time of inclusion, had a higher socioeconomic index, were more educated, had higher BMI, were younger at menarche, more likely to be premenopausal at baseline and more commonly users of oral contraceptives and hormone replacement therapy (Table 1).

**Table 1:** Percentage distribution of cases and controls in relation to demographic, socio-economic, life-style factors, reproductive history and season

	Cases and controls			
	Cases (*n* = 1186)	Controls (*n* = 1186)	No Breast Cancer (*n* = 15,608)	Total (*n* = 17,035)
Age				
< 50	25.8	19.2	24.6	24.8
50–55	23.5	23.5	18.8	19.1
55–60	19.2	20.9	16.6	16.8
≥ 60	31.5	36.3	40.0	39.2
Socio-economic index				
Manual	33.2	40.4	38.2	37.7
Non-manual	61.0	52.8	53.2	53.7
Employer	5.7	6.9	7.6	7.5
Education				
O-level college	66.8	71.3	69.9	69.6
A-level college	6.9	7.8	7.0	6.9
University	26.3	20.9	22.9	23.2
Married or cohabiting				
No	32.9	32.1	33.3	33.2
Yes	67.1	67.9	66.7	66.8
Parity				
1	19.0	20.8	21.6	21.4
2	45.3	41.0	40.7	41.0
3	15.1	16.9	16.7	16.5
4 or more	4.6	5.6	6.7	6.6
Nullipara	14.0	12.0	12.7	12.8
Missing	2.0	3.6	1.7	1.7
Age at first childbirth				
≤ 20	15.7	17.5	16.7	16.6
21–25	34.7	34.7	35.2	35.0
26–30	23.7	23.4	24.4	24.4
≥ 31	9.8	8.9	9.3	9.4
Age menarche				
≤ 12	23.7	20.7	21.8	21.9
13–14	53.1	53.0	52.9	52.9
≥ 15	23.2	26.3	24.6	24.5
Ever use of oral contraceptives				
No	45.9	52.2	51.3	50.9
Yes	54.1	47.8	48.6	49.0
Menopausal status				
Pre	30.1	22.2	25.6	26.1
Peri	8.5	7.8	6.9	7.0
Post	61.4	70.0	67.5	66.9
Oophorectomy, bilateral				
No	98.4	98.5	98.5	98.5
Yes	1.6	1.5	1.5	1.5
Hormone replacement therapy, current				
No	73.6	81.4	82.8	82.0
Yes	26.4	18.6	17.2	18.0
Alcohol consumption (g/d)				
0	5.4	8.2	7.8	7.6
< 15	63.3	64.4	64.1	64.0
15–30	15.1	13.1	13.9	14.0
≥ 30	4.3	2.2	2.2	2.4
Infrequent	11.9	12.1	11.9	11.9
Smoking				
Never	41.8	44.8	44.4	44.2
Current	28.0	26.5	28.0	28.0
Ex	30.2	28.7	27.5	27.7
Bode mass index (kg/m^2^)				
< 20	4.6	5.5	5.7	5.7
20–25	46.5	49.0	47.6	47.5
25–30	34.7	34.1	32.9	33.1
≥ 30	14.3	11.5	13.6	13.6
Season of collection of dietary data				
January-March	22.7	27.1	22.4	22.4
April-June	29.1	25.5	29.3	29.3
July–September	16.5	13.6	14.7	14.9
October-December	31.7	33.8	33.5	33.4

All data are presented as column percentage. Missing data ≤ 1% are not shown. Adapted from Sandsveden et al. (2017)

Women with low dietary intake of selenium were younger. In addition, women in the lowest and the highest quartiles of selenium intake had a higher educational level, were more often not married or cohabiting, were more frequent users of oral contraceptives and had lower BMI. The dietary assessment collected during spring tended to have a higher selenium level (Supplementary table S1). The results were similar when the same comparison was made between quartiles and potential confounders in the full cohort (Supplementary table S2).

### Dietary intake of selenium and breast cancer risk

No overall association between dietary intake of selenium and breast cancer risk was found (Tables 2, 3). In the case–control analysis, the adjusted OR for breast cancer in selenium intake for Q4 versus Q1 was 0.97 (0.76–1.23) (Table 2). Similarly, in the Cox-analysis, the adjusted RR for breast cancer in selenium intake for Q4 was 0.96 (0.83–1.12) versus Q1 (*P*_trend_ = 0.65) (Table 3).

**Table 2:** Odds ratio (OR) for cases and controls in relation to quartiles of selenium intake as compared to the first quartile and a merge of quartile 2 and 3, overall and stratified for body mass index (BMI)

Quartile	Median^a^ (ug/day)	All			BMI ≤ 25 kg/m^2^			BMI > 25 kg/m^2^		
		Cases/controls	Crude OR (95 CI^b^)	Adjusted^c^ OR (95 CI)	Cases/controls	Crude OR (95 CI)	Adjusted^d^ OR (95 CI)	Cases/controls	Crude OR (95 CI)	Adjusted^d^ OR (95 CI)
1	25.2	319/274	1.00	1.00	172/169	1.00	1.00	147/105	1.00	1.00
2	30.9	280/313	0.77 (0.61–0.97)	0.87 (0.68–1.10)	141/169	0.82 (0.60–1.12)	0.97 (0.69–1.35)	139/144	0.69 (0.49–0.97)	0.77 (0.54–1.11)
3	39.4	287/306	0.81 (0.64–1.01)	0.88 (0.69–1.12)	129/146	0.87 (0.63–1.19)	0.97 (0.68–1.36)	158/160	0.71 (0.50–0.98)	0.78 (0.55–1.12)
4	73.9	300/293	0.88 (0.70–1.11)	0.97 (0.76–1.23)	163/161	1.00 (0.73–1.34)	1.20 (0.86–1.68)	137/131	0.75 (0.53–1.06)	0.78 (0.54–1.14)
1	25.2	319/274	1.27 (1.04–1.55)	1.15 (0.93–1.41)	172/169	1.19 (0.91–1.55)	1.04 (0.77–1.38)	147/105	1.43 (1.07–1.93)	1.29 (0.94–1.77)
2 + 3	35.0	567/619	1.00	1.00	270/315	1.00	1.00	297/304	1.00	1.00
4	73.9	287/306	1.12 (0.92–1.36)	1.11 (0.90–1.36)	129/146	1.18 (0.90–1.55)	1.24 (0.93–1.67)	158/160	1.07 (0.80–1.43)	1.01 (0.74–1.37)

^a^Residuals of selenium intake quartiles are presented as the median of total dietary intake of selenium

^b^Confidence interval

^c^Adjusted for age, socioeconomic index, education, marriage, number of children, age at first childbirth, age at menarche, use of oral contraceptives, hormone replacement therapy, menopausal status, oophorectomy, smoking, BMI, alcohol consumption, season and year of inclusion

^d^Adjusted for all factors above except BMI. One control was missing BMI data

**Table 3:** Breast cancer incidence in the full cohort in relation to selenium levels, overall and stratified for body mass index (BMI)

Quartile	Median^a^ (ug/day)	Individuals (n)	Breast cancer cases (n)	Person-years	Incidence/ 100,000	All		BMI ≤ 25 kg/m^2^		BMI > 25 kg/m^2^	
						RR (95 CI^b^)	RR^c^ (95 CI)	RR (95 CI)	RR^d^ (95 CI)	RR (95 CI)	RR^d^ (95 CI)
1	24.7	4,259	374	87,302	428	1.00	1.00	1.00	1.00	1.00	1.00
2	30.8	4,258	341	86,359	395	0.92 (0.80–1.07)	0.91 (0.79–1.06)	1.02 (0.84–1.25)	1.01 (0.83–1.24)	0.81 (0.65–1.00)	0.82 (0.66–1.01)
3	39.0	4,260	343	85,774	400	0.94 (0.81–1.08)	0.91 (0.79–1.06)	0.94 (0.76–1.16)	0.91 (0.74–1.13)	0.90 (0.73–1.10)	0.90 (0.73–1.11)
4	72.8	4,258	369	85,149	433	1.01 (0.88–1.17)	0.96 (0.83–1.12)	1.06 (0.87–1.29)	1.00 (0.82–1.23)	0.96 (0.77–1.18)	0.93 (0.75–1.15)
P-trend						0.82	0.65	0.77	0.81	0.95	0.71
1	24.7	4,259	374	87,302	428	1.08 (0.95–1.22)	1.10 (0.96–1.24)	1.02 (0.86–1.21)	1.04 (0.87–1.24)	1.17 (0.98–1.41)	1.17 (0.97–1.40)
2 + 3	34.8	8,518	684	172,133	397	1.00	1.00	1.00	1.00	1.00	1.00
4	72.8	4,258	369	85,149	433	1.09 (0.96–1.24)	1.06 (0.93–1.20)	1.08 (0.90–1.28)	1.04 (0.87–1.25)	1.12 (0.93–1.35)	1.08 (0.90–1.30)

^a^Residuals of selenium intake quartiles are presented as the median of total dietary intake of selenium

^b^Confidence interval

^c^Adjusted for age, socioeconomic index, education, marriage, number of children, age at first childbirth, age at menarche, use of oral contraceptives, hormone replacement therapy, menopausal status, oophorectomy, smoking, BMI, alcohol consumption, season and year of inclusion

^d^Adjusted for all factors above except BMI. Twenty-six controls were missing BMI data

When the data were stratified for BMI, no association between dietary intake of selenium and breast cancer risk was found (Tables 2, 3). Furthermore, no overall association was seen between breast cancer risk and selenium intake using a reference category consisting of Q2 and Q3 (Tables 2, 4).

**Table 4:** Odds ratio (OR) for cases and controls in relation to quartiles selenium intake as compared to the first quartile and a merge of quartile 2 and 3, overall and stratified for smoking habits

Quartile	Median^a^ (ug/day)	Never smoker			Current smoker			Ex-smoker		
		Cases/ controls	Crude OR (95 CI^b^)	Adjusted^c^ OR (95 CI)	Cases/ controls	Crude OR (95 CI)	Adjusted^c^ OR (95 CI)	Cases/ controls	Crude OR (95 CI)	Adjusted^c^ OR (95 CI)
1	25.2	136/135	1.00	1.00	103/68	1.00	1.00	80/71	1.00	1.00
2	30.9	122/140	0.87 (0.62–1.22)	0.98 (0.68–1.42)	69/88	0.52 (0.33–0.80)	0.57 (0.35–0.94)	89/85	0.93 (0.60–1.44)	1.21 (0.74–1.99)
3	39.4	125/128	0.97 (0.69–1.37)	1.01 (0.69–1.46)	75/95	0.52 (0.34–0.80)	0.54 (0.33–0.88)	87/82	0.92 (0.61–1.46)	1.27 (0.77–2.08)
4	73.9	113/128	0.88 (0.62–1.24)	1.01 (0.69–1.48)	85/63	0.89 (0.57–1.39)	0.92 (0.55–1.53)	102/102	0.89 (0.58–1.35)	1.06 (0.66–1.70)
1	25.2	136/135	1.09 (0.81–1.47)	1.01 (0.73–1.39)	103/68	1.93 (1.32–2.80)	1.81 (1.18–2.77)	80/71	1.07 (0.73–1.57)	0.81 (0.52–1.25)
2 + 3	35.0	247/268	1.00	1.00	144/183	1.00	1.00	176/167	1.00	1.00
4	73.9	125/128	0.96 (0.71–1.30)	1.01 (0.73–1.42)	75/95	1.72 (1.16–2.54)	1.65 (1.06–2.58)	87/82	0.95 (0.67–1.34)	0.86 (0.58–1.26)

^a^Residuals of selenium intake quartiles are presented as the median of total dietary intake of selenium

^b^Confidence interval

^c^Adjusted for age, socioeconomic index, education, marriage, number of children, age at first childbirth, age at menarche, use of oral contraceptives, hormone replacement therapy, menopausal status, oophorectomy, body mass index, alcohol consumption, season and year of inclusion. One control was missing smoking data

In the Cox-analysis, no association between selenium intake and breast cancer risk was found when the data were stratified for smoking status (Supplementary table S4). However, in the case–control study, an association was seen in the group of current smokers (Table 4). The association formed a U-curve, with adjusted OR in Q1: 1.00, Q2: 0.57 (0.35–0.94), Q3: 0.54 (0.33–0.88) and Q4: 0.92 (0.55–1.53) (Table 4). When merging Q2 and Q3 the adjusted OR for Q1 vs Q2 + Q3 was 1.81 (1.18–2.77) and for Q4 vs Q2 + Q3 it was 1.65 (1.06–2.58) (Table 4).

When selenium intake was analyzed as a continuous variable in the case–control study, the adjusted OR was 1.01 (0.97–1.04).

In the Cox-analysis, no association between selenium intake ≤ 55 ug/day compared to selenium intake > 55 ug/day and breast cancer risk was seen. The adjusted RR for breast cancer in the low selenium intake group compared to the recommended selenium intake group was 0.99 (0.86–1.12) (Supplementary Table S3).

When repeating the analyses using total dietary intake of selenium adjusted for total energy intake in the multivariate logistic analysis, no overall association between dietary intake of selenium and breast cancer risk was found; the adjusted OR in Q1: 1.00 Q2: 0.84 (0.65–1.08), Q3: 0.86 (0.67–1.12) and Q4: 0.92 (0.71–1.19). Moreover, all results were similar in the sensitivity analyses with the additional adjustment for interviewer and dietary method; the adjusted OR for breast cancer in selenium intake for Q4 versus Q1 was 0.96 (0.75–1.23). Finally, the results were not significantly altered when cases during the first two years following baseline were excluded; the adjusted ORs of breast cancer for women in increasing quartiles of selenium intake were 1.00, 0.82 (0.61–1.10), 0.87 (0.65–1.17) and 0.94 (0.69–1.28).

### Serum selenium as a biomarker for dietary intake of selenium

In a crosstabulation, dietary intake of selenium and serum selenium were compared (Table 5). Serum selenium was higher in the group with high selenium intake (*n* = 217) than the group with low selenium intake (*n* = 191). However, the kappa value, 0.096 (*p* = 0.001), showed poor agreement between serum selenium and dietary intake of selenium. Likewise, when serum selenium and non-adjusted values of selenium intake were analyzed in a cross table, the kappa value was 0.096 (*p* = 0.001) (Supplementary Table S5).

**Table 5:** Serum selenium and dietary intake of selenium

(*n* = 593)	Median^a^ (ug/day)	Serum selenium					Total
		1 (*n* = 535)	2 (*n* = 522)	3 (*n* = 532)	4 (*n* = 521)	Missing	
		≤ 81.1 ng/ml	81.1—90.5 ng/ml	90.6 – 100 ng/ml	≥ 100 ng/ml	(*n* = 262)	
Dietary intake of selenium							
1	25.2	191 (32.2)	157 (26.5)	97 (16.4)	77 (13.0)	71 (12.0)	593 (100.0)
2	30.9	148 (25.0)	141 (23.8)	135 (17.9)	106 (10.6)	63 (10.6)	593 (100.0)
3	39.4	112 (18.9)	130 (21.9)	156 (26.3)	136 (22.9)	59 (9.9)	593 (100.0)
4	73.9	76 (12.8)	105 (17.7)	126 (21.2)	217 (36.6)	69 (11.6)	593 (100.0)
Total	(*n* = 2,372)	527 (22.2)	533 (22.5)	514 (21.7)	536 (22.6)	262 (11.0)	2,372 (100.0)

^a^Residuals of selenium intake quartiles are presented as the median of total dietary intake of selenium

The data shown in brackets are presented as row percentage

### Dietary intake of selenium, serum selenium and breast cancer risk

When the dietary intake of selenium and serum selenium were analyzed together in a logistic regression analysis, no association with breast cancer risk was seen (Table 6). When comparing the women with missing serum values with the lowest group, the adjusted OR was 0.86 (0.63–1.18) (Table 6).

**Table 6:** Odds ratio (OR) for cases and controls in relation to serum selenium levels and dietary intake of selenium

Group^a^		Case/controls	Crude OR (CI^b^ 95)	Adjusted OR^c^ (CI 95)
1	Low serum levels + low dietary intake	338/297	1.00	1.00
2	High serum levels + low dietary intake	192/225	0.75 (0.59–0.96)	0.81 (0.62–1.06)
3	Low serum levels + high dietary intake	202/220	0.81 (0.63–1.03)	0.87 (0.67–1.12)
4	High serum levels + high dietary intake	324/312	0.91 (0.73–1.14)	0.97 (0.76–1.23)
	Missing vs group 1	130/132	0.87 (0.65–1.15)	0.86 (0.63–1.18)

^a^Groups of serum selenium and selenium intake with low defined as a merge of Q1 and Q2 and high defined as a merge of Q3 and Q4. Serum selenium quartiles and quartiles of selenium intake are presented in Table 4

^b^Confidence interval

^c^Adjusted for energy intake, age, socioeconomic index, education, marriage, number of children, age at first childbirth, age at menarche, use of oral contraceptives, hormone replacement therapy, menopausal status, oophorectomy, smoking, body mass index, alcohol consumption, season and year of inclusion

## Discussion

The primary objective of this study was to investigate whether dietary intake of selenium was associated with risk of developing breast cancer. We did not find any overall association between selenium intake and breast cancer risk. No association between breast cancer and a combination of dietary intake of selenium and serum selenium was seen. Poor agreement was shown between serum selenium and dietary intake of selenium.

Previous investigations of the association between selenium and breast cancer risk have shown inconsistent results. A meta-analysis by Cai et al. (2016) indicated that high serum selenium, toenail selenium, dietary intake of selenium and breast tissue selenium were associated with a lower risk of breast cancer (pooled OR: 0.88; 95% CI:0.84–0.93) [5]. This is in line with another recent prospective meta-analysis by Kuria et al. (2018) on selenium in the diet and supplements and overall cancer risk. However, the metanalysis by Kuria et al. showed no evidence of high selenium intake and breast cancer-specific incidence [6]. In addition, the meta-analysis by Cai et al. had very high weight (79%) in a small retrospective study and also included studies with both cancer incidence and cancer mortality as endpoints, making it problematic to interpret the results [5]. In contrast, our results from a previous study on pre-diagnostic serum selenium and breast cancer risk [2], as well as a Cochrane review by Vinceti et al. (2018) [3] and a prospective cohort study on selenium intake and breast cancer [9], showed no relationship between selenium and breast cancer risk.

Another way of investigating the potential cancer preventing effect of selenium is to implement RCTs with selenium as supplementation. Vinceti et al. (2018) included 11 RCTs involving 44,743 participants, 94% men, randomized to either selenium supplements or placebo. The results showed no useful effect of selenium supplementation in reducing overall cancer risk [3]. The review involved only one RCT with breast cancer incidence as a primary outcome. The trial was conducted in Poland in 2011 and included 1,135 women carrying the breast cancer-associated mutation *BRCA1.* The final analysis was found on 105 incident cases and showed no decreased cancer risk by selenium supplementation [7]. Nevertheless, some RCTs even indicate that selenium supplementation could potentially increase the risk of some type of cancer, e.g. high-grade prostate cancer [3, 5, 37]. High supplemental intake has also been linked to a statistically non-significant increase in type II diabetes [38] and an increase in all-cause mortality [39].

One of the findings of our study was the discovery of a U-shaped association in the logistic regression model between quartiles of selenium intake and breast cancer risk in the group of current smokers. Walters and Chiang (2018) discuss in a review their theory of a U-shaped dose–response relationship in selenium-supplemented dogs, which parallels previous studies on selenium status and prostate cancer risk in humans. In other words, they suggest that reaching a mid-range selenium status is better than being too low or too high [18]. The U-shaped health effects of selenium were also discussed by Professor Rayman in her Lancet article, where she suggested that selenium supplements might be favorable for people with low status, while those with medium or high status should not take selenium supplements because of the potential oppositional effect [11].

Since most of the abovementioned studies were conducted on men, and mostly investigating selenium and prostate cancer risk, it is relevant to know if there are any differences in the anticancerogenic effects of selenium between men and women. A review by Walters et al. (2004) evaluated the possibility of differences in the anticarcinogenic effects in humans based on sex and concluded that high overall cancer risk in men might be more strongly associated with low selenium status than cancer risk in women, even though more research is needed [40]. However, our study suggests a protective effect of selenium on breast cancer in women in the group of current smokers.

Some observational studies on prostate cancer and selenium also suggest an effect modification by smoking, showing a stronger inverse association among smokers [41–43]. One potential explanation for this result may be that smoking results in an increased production of reactive oxygen species (ROS) [44], while several selenoenzymes reduce the damaging effect of ROS on DNA [13]. Furthermore, the protective role of selenium in smokers could also be explained by the occurrence of oxidative response elements in the promotor regions of some selenoenzymes, e.g. glutathione peroxidase [45], which can increase the transcription of these selenoenzymes related to exposure of oxidative stress [46].

This study has several strengths. The large number of subjects gives the study good statistical power. Moreover, the dietary assessment method showed good ranking compared to a reference method [30], and the residual method adjusts for the differences in total energy intake. Additionally, the inclusion of both dietary data and serum is unique, and our study measures dietary intake of selenium pre-diagnostically. The adjustment for confounding factors such as socioeconomic factors and lifestyle factors is an additional strength.

The overall completeness and validity of the Swedish Cancer Registry, which we used to select our cases, is high and equivalent to other high-quality registers in Northern Europe [47]. The MDCS, with an overall participation rate of 40.8%, had the same sociodemographic structure and prevalence of smoking and obesity as a mailed health survey with a participation rate of 74.6% [27]. A subject was only regarded as a complete participant if she completed the questionnaire, the dietary assessment and the anthropometric measurements [27]. Due to this, all our cases and controls had data on dietary intake of selenium.

Potential weaknesses of the study are the fact that different selection criteria, both matched and randomly selected, were used for the collection of our control groups. However, the different selection criteria did not influence the results considerably, as described in an earlier study, when OR was calculated for the exact same cases and controls in relation to quartiles of serum selenium stratified for the different control groups [2]. In addition, dietary intake as an exposure variable has some methodological difficulties which makes it challenging to obtain adequate information. For example, one problem is the fact that information on dietary habits usually covers a limited period of a person's life. Furthermore, nutrients are not consumed in isolation and it is difficult to separate the effects of specific nutrients [48]. Thus, it is difficult to adjust for all the potential confounding factors, and though we tried to adjust for the most important ones, residual confounding is an issue.

We found a poor agreement between serum selenium and dietary intake of selenium. The complex association between determinants of selenium and serum selenium concentration has not yet been fully identified [20]. Dietary assessment, in contrast to serum selenium, determines the selenium status before any metabolic conversion and distribution within the body has been made [49]. In addition, the distribution of selenium in different body compartments relies on the specific chemical species of the ingested selenium and the intake of other dietary factors such as cadmium which may change the storage of ingested selenium in the body by modifying its bioavailability or increasing its excretion [49]. On the other hand, the precision of dietary assessment methods can be affected by the fact that the selenium content in certain food groups can vary even within a small region and is dependent on from where the food products have been imported from [10]. Consequently, it is important to highlight the potential exposure misclassification of observational epidemiologic studies based on overall selenium content in dietary intake or serum.

Additional studies are necessary on breast cancer and selenium status to confirm the possible effect modification caused by smoking. In addition, better biomarkers are needed for measuring selenium status when studying cancer risk as the primary health outcome.

## Conclusions

The present cohort study included 1,427 incident breast cancer cases and found no overall association between dietary intake of selenium and breast cancer risk. No overall association was found when combinations of dietary and serum selenium were compared to breast cancer risk. Finally, poor agreement was shown between serum selenium and dietary intake of selenium.

## Supplementary Information

Below is the link to the electronic supplementary material.

Supplementary file1 (docx 22 kb)

Supplementary file2 (docx 25 kb)

Supplementary file3 (docx 14 kb)

Supplementary file4 (docx 17 kb)

Supplementary file5 (docx 15 kb)

## Data Availability

The data will be shared on reasonable request to the corresponding author.
