# Supplementary material for: Risk of breast cancer in relation to dietary intake of selenium and serum selenium as a marker of dietary intake: a prospective cohort study within The Malmö Diet and Cancer Study
Source: Cancer Causes Control. 2021 Apr 29;32(8):815–26. doi: 10.1007/s10552-021-01433-1 (PMC8236480; doi:10.1007/s10552-021-01433-1)
Supplement: Supplementary file 1 — Supplementary file1 (docx 22 kb) [file 10552_2021_1433_MOESM1_ESM.docx]

Supplementary table S1. Percentage distribution of selenium intake quartiles in relation to demographic, socio-economic, life-style factors, reproductive history and season

|  | | Selenium intake^a^ | | | | |
| --- | --- | --- | --- | --- | --- | --- |
|  |  | 1 (n=593) | 2 (n=593) | 3 (n=593) | 4 (n=593) | Total |
|  |  | 25.2 ug/day | 30.9 ug/day | 39.4 ug/day | 73.9 ug/day | (n=2372) |
| Age | <50 | 31.4 | 23.3 | 19.2 | 16.2 | 22.2 |
|  | 50-55 | 23.3 | 22.9 | 22.6 | 25.3 | 23.5 |
|  | 55-60 | 16.4 | 19.9 | 20.9 | 23.1 | 20.1 |
|  | ≥60 | 29.0 | 33.9 | 37.3 | 35.4 | 33.9 |
| Socio-economic index | Manual | 37.3 | 39.7 | 38.5 | 31.7 | 36.8 |
|  | Non-manual | 55.9 | 55.5 | 54.8 | 61.3 | 56.9 |
|  | Employer | 6.8 | 4.8 | 6.6 | 7.0 | 6.3 |
| Education | O-level college | 67.9 | 71.7 | 71.7 | 64.9 | 69.0 |
|  | A-level college | 6.8 | 7.3 | 7.4 | 7.9 | 7.4 |
|  | University | 25.4 | 21.0 | 20.9 | 27.2 | 23.6 |
| Married or cohabiting | No | 34.6 | 30.9 | 27.2 | 37.4 | 32.5 |
|  | Yes | 65.4 | 69.1 | 72.8 | 62.6 | 67.5 |
| Parity | 1 | 19.9 | 21.4 | 20.1 | 18.2 | 19.9 |
|  | 2 | 44.4 | 40.0 | 42.3 | 45.9 | 43.1 |
|  | 3 | 16.2 | 17.2 | 15.2 | 15.5 | 16.0 |
|  | 4 or more | 5.2 | 5.7 | 6.6 | 3.0 | 5.1 |
|  | Nullipara | 13.3 | 12.1 | 13.3 | 13.2 | 13.0 |
|  | Missing | 1.0 | 3.5 | 2.5 | 4.2 | 2.8 |
| Age at first childbirth | ≤20 | 16.9 | 18.2 | 16.7 | 14.5 | 16.6 |
|  | 21-25 | 35.1 | 33.4 | 36.8 | 33.6 | 34.7 |
|  | 26-30 | 22.9 | 23.4 | 21.9 | 26.0 | 23.6 |
|  | ≥31 | 10.6 | 9.3 | 8.8 | 8.6 | 9.3 |
| Age at menarche | ≤12 | 23.3 | 21.4 | 23.0 | 21.3 | 22.2 |
|  | 13-14 | 50.9 | 52.5 | 54.3 | 54.4 | 53.0 |
|  | ≥15 | 25.7 | 26.1 | 22.8 | 24.3 | 24.7 |
| Ever use of oral contraceptives | No | 45.2 | 53.4 | 50.8 | 46.9 | 49.1 |
|  | Yes | 54.8 | 46.6 | 49.2 | 53.1 | 50.9 |
| Menopausal status | Pre | 32.9 | 26.5 | 23.6 | 21.6 | 26.1 |
|  | Peri | 9.4 | 7.9 | 7.1 | 8.3 | 8.2 |
|  | Post | 57.7 | 65.6 | 69.3 | 70.2 | 65.7 |
| Ooephorectomy, bilateral | No | 98.8 | 98.8 | 98.7 | 97.5 | 98.4 |
|  | Yes | 1.2 | 1.2 | 1.3 | 2.5 | 1.6 |
| Hormone replacement therapy, current | No | 77.0 | 80.8 | 78.6 | 73.7 | 77.5 |
|  | Yes | 23.0 | 19.2 | 21.4 | 26.3 | 22.5 |
| Alcohol consumption (g/d) | 0 | 7.8 | 8.1 | 7.3 | 4.0 | 6.8 |
|  | <15 | 59.9 | 65.4 | 64.1 | 66.1 | 63.9 |
|  | 15-30 | 14.5 | 10.8 | 14.8 | 16.2 | 14.1 |
|  | >30 | 4.2 | 2.2 | 3.5 | 3.0 | 3.2 |
|  | Infrequent | 13.7 | 13.3 | 10.3 | 10.6 | 12.0 |
| Smoking | Never | 45.7 | 44.2 | 42.7 | 40.6 | 43.3 |
|  | Current | 28.8 | 26.5 | 28.7 | 25.0 | 27.2 |
|  | Ex | 25.5 | 29.3 | 28.5 | 34.4 | 29.4 |
| Body mass index (kg‎/m²) | <20 | 6.6 | 3.0 | 4.0 | 6.4 | 5.0 |
|  | 20-25 | 50.9 | 49.2 | 42.3 | 48.4 | 47.7 |
|  | 25-30 | 30.2 | 33.9 | 39.6 | 33.7 | 34.4 |
|  | ≥30 | 12.3 | 13.8 | 14.0 | 11.5 | 12.9 |
| Season of collection of dietary data | January-March | 22.1 | 23.9 | 25.6 | 27.8 | 24.9 |
|  | April-June | 27.8 | 27.3 | 25.1 | 29.0 | 27.3 |
|  | July-September | 15.3 | 15.0 | 17.2 | 12.6 | 15.1 |
|  | October-December | 34.7 | 33.7 | 32.0 | 30.5 | 32.8 |

^a^Residuals of selenium intake quartiles are presented as the median of total dietary intake of selenium.

All data are presented as column percentage. Missing data ≤1% is not shown.
