# Supplementary material for: Risk of breast cancer in relation to dietary intake of selenium and serum selenium as a marker of dietary intake: a prospective cohort study within The Malmö Diet and Cancer Study
Source: Cancer Causes Control. 2021 Apr 29;32(8):815–26. doi: 10.1007/s10552-021-01433-1 (PMC8236480; doi:10.1007/s10552-021-01433-1)
Supplement: Supplementary file 2 — Supplementary file2 (docx 25 kb) [file 10552_2021_1433_MOESM2_ESM.docx]

Supplementary table S2. Percentage distribution of selenium intake quartiles in the full cohort in relation to demographic, socio-economic, life-style factors, reproductive history and season

|  | | Selenium intake^a^ | | | | |
| --- | --- | --- | --- | --- | --- | --- |
|  |  | 1 (n=4259) | 2 (n=4258) | 3 (n=4260) | 4 (n=4258) | Total |
|  |  | 24.7 ug/day | 30.8 ug/day | 39.0 ug/day | 72.8 ug/day | (n=17035) |
| Age | <50 | 32.1 | 24.8 | 21.7 | 20.7 | 24.8 |
|  | 50-55 | 20.3 | 18.4 | 19.3 | 18.6 | 19.1 |
|  | 55-60 | 16.2 | 17.3 | 16.2 | 17.5 | 16.8 |
|  | ≥60 | 31.3 | 39.5 | 42.8 | 43.2 | 39.2 |
| Socio-economic index | Manual | 41.0 | 40.4 | 37.1 | 32.5 | 37.7 |
|  | Non-manual | 50.4 | 51.7 | 54.6 | 58.2 | 53.7 |
|  | Employer | 7.6 | 6.5 | 7.5 | 8.4 | 7.5 |
|  | Missing | 1.0 | 1.4 | 0.8 | 0.9 | 1.0 |
| Education | O-level college | 70.8 | 72.3 | 70.3 | 65.1 | 69.6 |
|  | A-level college | 7.3 | 6.9 | 6.7 | 6.9 | 6.9 |
|  | University | 21.6 | 20.5 | 22.7 | 27.8 | 23.2 |
| Married or cohabiting | No | 35.1 | 30.4 | 31.4 | 35.9 | 33.2 |
|  | Yes | 64.9 | 69.5 | 68.6 | 64.1 | 66.8 |
| Parity | 1 | 21.6 | 21.4 | 21.5 | 20.9 | 21.4 |
|  | 2 | 41.8 | 41.1 | 40.3 | 40.9 | 41.0 |
|  | 3 | 17.0 | 16.0 | 17.0 | 16.0 | 16.5 |
|  | 4 or more | 6.6 | 7.2 | 6.3 | 6.2 | 6.6 |
|  | Nullipara | 11.6 | 12.4 | 13.3 | 14.0 | 12.8 |
|  | Missing | 1.4 | 1.8 | 1.6 | 2.1 | 1.7 |
| Age at first childbirth | ≤20 | 17.6 | 17.2 | 16.6 | 15.1 | 16.6 |
|  | 21-25 | 35.3 | 35.4 | 34.5 | 34.9 | 35.0 |
|  | 26-30 | 24.1 | 24.2 | 24.4 | 24.7 | 24.4 |
|  | ≥31 | 9.9 | 8.9 | 9.6 | 9.1 | 9.4 |
| Age at menarche | ≤12 | 21.6 | 20.5 | 22.4 | 23.1 | 21.9 |
|  | 13-14 | 52.2 | 53.9 | 52.7 | 52.9 | 52.9 |
|  | ≥15 | 25.6 | 24.7 | 24.2 | 23.5 | 24.5 |
| Ever use of oral contraceptives | No | 47.1 | 51.6 | 52.9 | 51.8 | 50.9 |
|  | Yes | 52.7 | 48.3 | 47.0 | 48.1 | 49.0 |
| Menopausal status | Pre | 32.0 | 25.8 | 23.7 | 23.0 | 26.1 |
|  | Peri | 8.1 | 6.5 | 6.5 | 7.0 | 7.0 |
|  | Post | 59.9 | 67.8 | 69.8 | 70.0 | 66.9 |
| Ooephorectomy, bilateral | No | 98.8 | 98.4 | 98.5 | 98.2 | 98.5 |
|  | Yes | 1.2 | 1.6 | 1.5 | 1.8 | 1.5 |
| Hormone replacement therapy, current | No | 82.9 | 83.1 | 82.5 | 78.6 | 81.8 |
|  | Yes | 16.8 | 16.6 | 17.1 | 21.3 | 18.0 |
| Alcohol consumption (g/d) | 0 | 8.4 | 8.1 | 7.6 | 6.4 | 7.6 |
|  | <15 | 62.2 | 65.6 | 65.1 | 63.1 | 64.0 |
|  | 15-30 | 13.1 | 12.5 | 13.5 | 16.8 | 14.0 |
|  | >30 | 2.3 | 1.8 | 2.3 | 2.9 | 2.4 |
|  | Infrequent | 13.8 | 11.9 | 11.2 | 10.7 | 11.9 |
| Smoking | Never | 42.6 | 46.3 | 44.9 | 42.9 | 44.2 |
|  | Current | 31.9 | 26.9 | 27.0 | 26.4 | 28.0 |
|  | Ex | 25.5 | 26.7 | 28.1 | 30.7 | 27.7 |
| Body mass index (kg‎/m²) | <20 | 7.4 | 4.8 | 4.5 | 6.0 | 5.7 |
|  | 20-25 | 51.0 | 46.1 | 43.8 | 49.3 | 47.5 |
|  | 25-30 | 29.8 | 34.2 | 35.9 | 32.5 | 33.1 |
|  | ≥30 | 11.6 | 14.9 | 15.7 | 12.1 | 13.6 |
| Season of collection of dietary data | January-March | 21.5 | 21.3 | 22.3 | 24.6 | 22.4 |
|  | April-June | 28.1 | 28.2 | 29.7 | 31.2 | 29.3 |
|  | July-September | 15.5 | 15.4 | 16.2 | 12.6 | 14.9 |
|  | October-December | 34.9 | 35.2 | 31.8 | 31.6 | 33.4 |

^a^Residuals of selenium intake quartiles are presented as the median of total dietary intake of selenium.

All data are presented as column percentage. Missing data ≤1% is not shown.
