# Supplementary material for: Risk of breast cancer in relation to dietary intake of selenium and serum selenium as a marker of dietary intake: a prospective cohort study within The Malmö Diet and Cancer Study
Source: Cancer Causes Control. 2021 Apr 29;32(8):815–26. doi: 10.1007/s10552-021-01433-1 (PMC8236480; doi:10.1007/s10552-021-01433-1)
Supplement: Supplementary file 3 — Supplementary file3 (docx 14 kb) [file 10552_2021_1433_MOESM3_ESM.docx]

| Group | Selenium intake (ug/day) | Individuals (n) | Breast cancer cases (n) | Person-years | Incidence/  100,000 | RR (95 CI^a^) | RR^d^ (95 CI) | |
| --- | --- | --- | --- | --- | --- | --- | --- | --- |
| Low | ≤55 | 13562 | 1127 | 274,620 | 410 | 0.96 (0.84-1.09) | | 0.99 (0.86-1.12) |
| RDI | >55 | 3473 | 300 | 69,963 | 428 | 1.00 | | 1.00 |

Supplementary table S3. Breast cancer incidence in the full cohort in relation to recommended dietary intake (RDI) of 55 ug/day

^a^Confidence interval

^b^Adjusted for energy intake, age, socioeconomic index, education, marriage, number of children, age at first childbirth, age at menarche, use of oral contraceptives, hormone replacement therapy, menopausal status, oophorectomy, smoking, BMI, alcohol consumption, season and year of inclusion.
