# Supplementary material for: Risk of breast cancer in relation to dietary intake of selenium and serum selenium as a marker of dietary intake: a prospective cohort study within The Malmö Diet and Cancer Study
Source: Cancer Causes Control. 2021 Apr 29;32(8):815–26. doi: 10.1007/s10552-021-01433-1 (PMC8236480; doi:10.1007/s10552-021-01433-1)
Supplement: Supplementary file 4 — Supplementary file4 (docx 17 kb) [file 10552_2021_1433_MOESM4_ESM.docx]

| Quartile | Median^a^ (ug/day) | Individuals (n) | Breast cancer cases (n) | Person-years | Incidence  /100,000 | Never smoker | |  | Current smoker | |  | Ex-smoker | |
| --- | --- | --- | --- | --- | --- | --- | --- | --- | --- | --- | --- | --- | --- |
|  |  |  |  |  |  | RR (95 CI^b^) | RR^c^ (95 CI) |  | RR (95 CI) | RR^d^ (95 CI) |  | RR (95 CI) | RR^d^ (95 CI) |
| 1 | 24.7 | 4259 | 374 | 87,302 | 428 | 1.00 | 1.00 |  | 1.00 | 1.00 |  | 1.00 | 1.00 |
| 2 | 30.8 | 4258 | 341 | 86,359 | 395 | 0.88 (0.70-1.10) | 0.86 (0.69-1.08) |  | 0.88 (0.67-1.15) | 0.85 (0.65-1.13) |  | 0.61 (0.80-1.40) | 1.05 (0.80-1.39) |
| 3 | 39.0 | 4260 | 343 | 85,774 | 400 | 0.93 (0.74-1.16) | 0.90 (0.72-1.13) |  | 0.96 (0.74-1.26) | 0.94 (0.71-1.23) |  | 0.94 (0.71-1.24) | 0.91 (0.69-1.22) |
| 4 | 72.8 | 4258 | 369 | 85,149 | 433 | 0.90 (0.72-1.13) | 0.89 (0.70-1.12) |  | 1.02 (0.78-1.33) | 0.95 (0.72-1.25) |  | 1.17 (0.90-1.53) | 1.11 (0.85-1.45) |
| P-trend |  |  |  |  |  | 0.49 | 0.39 |  | 0.80 | 0.83 |  | 0.35 | 0.66 |
| 1 | 24.7 | 4259 | 374 | 87,302 | 428 | 1.11 (0.92-1.35) | 1.14 (0.94-1.38) |  | 1.09 (0.87-1.37) | 1.12 (0.89-1.41) |  | 1.00 (0.79-1.28) | 1.02 (0.80-1.30) |
| 2+3 | 34.8 | 8518 | 684 | 172,133 | 397 | 1.00 | 1.00 |  | 1.00 | 1.00 |  | 1.00 | 1.00 |
| 4 | 72.8 | 4258 | 369 | 85,149 | 433 | 1.00 (0.82-1.23) | 1.01 (0.82-1.24) |  | 1.11 (0.87-1.41) | 1.06 (0.83-1.36) |  | 1.18 (0.94-1.47) | 1.13 (0.90-1.41) |

Supplementary table S4. Breast cancer incidence in the full cohort in relation to selenium levels, stratified for smoking habits

^a^Residuals of selenium intake quartiles are presented as the median of total dietary intake of selenium.

^b^Confidence interval

^c^Adjusted for age, socioeconomic index, education, marriage, number of children, age at first childbirth, age at menarche, use of oral contraceptives, hormone replacement therapy, menopausal status, oophorectomy, body mass index, alcohol consumption, season and year of inclusion. Six controls were missing smoking data.
