# Supplementary material for: Risk of breast cancer in relation to dietary intake of selenium and serum selenium as a marker of dietary intake: a prospective cohort study within The Malmö Diet and Cancer Study
Source: Cancer Causes Control. 2021 Apr 29;32(8):815–26. doi: 10.1007/s10552-021-01433-1 (PMC8236480; doi:10.1007/s10552-021-01433-1)
Supplement: Supplementary file 5 — Supplementary file5 (docx 15 kb) [file 10552_2021_1433_MOESM5_ESM.docx]

Supplementary table S5. Serum selenium and absolute dietary intake of selenium

|  | | | Serum selenium | | | | |  |
| --- | --- | --- | --- | --- | --- | --- | --- | --- |
|  |  |  |  |  |  |  |  |  |
|  | (n=593) | Quartiles (ug/day) | 1 (n=535) | 2 (n=522) | 3 (n=532) | 4 (n=521) | Missing | Total |
|  |  |  | ≤ 81.1 ng/ml | 81.1 - 90.5 ng/ml | 90.6 – 100 ng/ml | ≥ 100 ng/ml | (n=262) |  |
| Dietary intake of selenium | 1 | ≤28 | 189 (31.9) | 149 (25.1) | 105 (17.7) | 82 (13.8) | 68 (11.5) | 593 (100.0) |
|  |  |  |  |  |  |  |  |  |
|  | 2 | 28.1-35.9 | 141 (23.8) | 147 (24.8) | 137 (23.1) | 106 (17.9) | 62 (10.5) | 593 (100.0) |
|  |  |  |  |  |  |  |  |  |
|  | 3 | 36.0-50.1 | 119 (20.1) | 129 (21.8) | 160 (27.0) | 124 (20.9) | 61 (10.3) | 593 (100.0) |
|  |  |  |  |  |  |  |  |  |
|  | 4 | ≥59.2 | 86 (14.5) | 97 (16.4) | 130 (21.9) | 209 (35.2) | 71 (12.0) | 593 (100.0) |
|  |  |  |  |  |  |  |  |  |
|  | Total | (n=2372) | 535 (22.6) | 522 (22.0) | 532 (22.4) | 521 (22.0) | 262 (11.0) | 2372 (100.0) |

The data shown in brackets is presented as row percentage.
